# Metabolically healthy obesity is associated with higher risk of both hyperfiltration and mildly reduced estimated glomerular filtration rate: the role of serum uric acid in a cross-sectional study

**DOI:** 10.1186/s12967-023-04003-y

**Published:** 2023-03-23

**Authors:** Hong Zhang, Rui Chen, Xiaohong Xu, Minxing Yang, Wenrong Xu, Shoukui Xiang, Long Wang, Xiaohong Jiang, Fei Hua, Xiaolin Huang

**Affiliations:** 1 Department of Endocrine and Metabolic Diseases, The First People’s Hospital of Changzhou, Third Affiliated Hospital of Soochow University, 185 Juqianjie Road, Changzhou, 213000 Jiangsu China; 2 Department of Nephrology, Nanjing Drum Tower Hospital Group Suqian Hospital, Suqian, 223800 Jiangsu China; 3 Department of Nephrology, The Affiliated Suqian Hospital of Xuzhou Medical University, Suqian, 223800 Jiangsu China; 4 Department of Immunization Program, Liangxi District Center for Disease Control and Prevention, Wuxi, 214000 Jiangsu China

**Keywords:** Metabolically healthy obesity, Mildly reduced estimated glomerular filtration rate, Hyperfiltration, Uric acid

## Abstract

**Background:**

The impact of metabolically healthy obesity (MHO) on kidney dysfunction remains debatable. Moreover, few studies have focused on the early stages of kidney dysfunction indicated by hyperfiltration and mildly reduced eGFR. Thus, we aimed to investigate the association between the MHO and early kidney dysfunction, which is represented by hyperfiltration and mildly reduced estimated glomerular filtration rate (eGFR), and to further explore whether serum uric acid affects this association.

**Methods:**

This cross-sectional study enrolled 1188 residents aged ≥ 40 years old from Yonghong Communities. Metabolically healthy phenotypes were categorized based on Adult Treatment Panel III criteria. Obesity was defined as body mass index (BMI) ≥ 25 kg/m^2^. Mildly reduced eGFR was defined as being in the range 60 < eGFR ≤ 90 ml/min/1.73m^2^. Hyperfiltration was defined as eGFR > 95th percentile after adjusting for sex, age, weight, and height.

**Results:**

Overall, MHO accounted for 12.8% of total participants and 24.6% of obese participants. Compared to metabolically healthy non-obesity (MHNO), MHO was significantly associated with an increased risk of mildly reduced eGFR (odds ratio [OR] = 1.85, 95% confidence interval [CI]  1.13–3.01) and hyperfiltration (OR = 2.28, 95% CI 1.03–5.09). However, upon further adjusting for uric acid, the association between the MHO phenotype and mildly reduced eGFR was reduced to null. Compared with MHNO/non-hyperuricemia, MHO/non-hyperuricemia was associated with an increased risk of mildly reduced eGFR (OR = 2.04, 95% CI  1.17–3.58), whereas MHO/hyperuricemia was associated with an observably increased risk (OR = 3.07, 95% CI  1.34–7.01).

**Conclusions:**

MHO was associated with an increased risk of early kidney dysfunction, and the serum uric acid partially mediated this association. Further prospective studies are warranted to clarify the causality.

**Supplementary Information:**

The online version contains supplementary material available at 10.1186/s12967-023-04003-y.

## Background

Chronic kidney disease (CKD) has emerged as a critical public health problem, affecting approximately 10% of adults worldwide [1]. CKD may lead to end-stage renal disease (ESRD) and increased cardiovascular morbidity and mortality, and it is expected to be the fifth-leading cause of death by 2040 [2]. Therefore, early detection and prevention are of great significance to clinical practice. Glomerular hyperfiltration (GH), defined as an increased whole-kidney glomerular filtration rate (GFR) or an increased filtration per nephron [3], is considered an early and reversible stage of glomerular damage and a harbinger for subsequent development of CKD [4, 5]. Of note, a mildly reduced GFR, characterized as a GFR < 90 ml/min/1.73m^2^, is also an indication of early-stage CKD. Fox et al*.* report that patients with mildly reduced GFR, as assessed by estimated GFR (eGFR), were three times more likely to progress to CKD than those with normal GFR values [6].

Numerous studies have established that obesity is a risk factor for CKD [7, 8]. However, there has been no consensus as to whether the underlying mechanisms of obesity are definite risk factors for kidney dysfunction. Obesity is usually accompanied by metabolic abnormalities, including elevated blood glucose [9], elevated blood pressure [10], and lipid disorders [11]. Most studies declare that the metabolic abnormalities induced by obesity played a key role in kidney dysfunction [12, 13]. However, there is a unique subgroup of obese individuals who have a normal metabolic status, for instance, appropriate blood glucose and pressure levels and favorable lipid profiles. This obesity phenotype is termed metabolically healthy obesity (MHO) [14]. The impact of MHO on kidney dysfunction remains debatable. Some studies have reported that MHO is associated with a higher risk of incident CKD, suggesting that having a metabolically healthy status does not protect obese individuals from the onset of CKD [15, 16], but other studies have not found significant associations between MHO and incident CKD [17]. Furthermore, most studies have investigated the association between MHO and severe kidney disease, characterized as eGFR < 60 ml/min/1.73m^2^, whereas few studies have focused on the early stages of CKD indicated by hyperfiltration and mildly reduced eGFR.

Consequently, the main aim of the present study is to investigate the associations of MHO with hyperfiltration and mildly reduced eGFR, to clarify whether obesity without metabolic abnormalities is adversely associated with early kidney dysfunction. Additionally, we explore the possible mechanism between MHO and early kidney dysfunction. We hypothesize that individuals with MHO have a higher potential for early kidney dysfunction and that metabolic abnormalities only partially mediate this association.

## Methods

### Study population

**Fig. 1:**
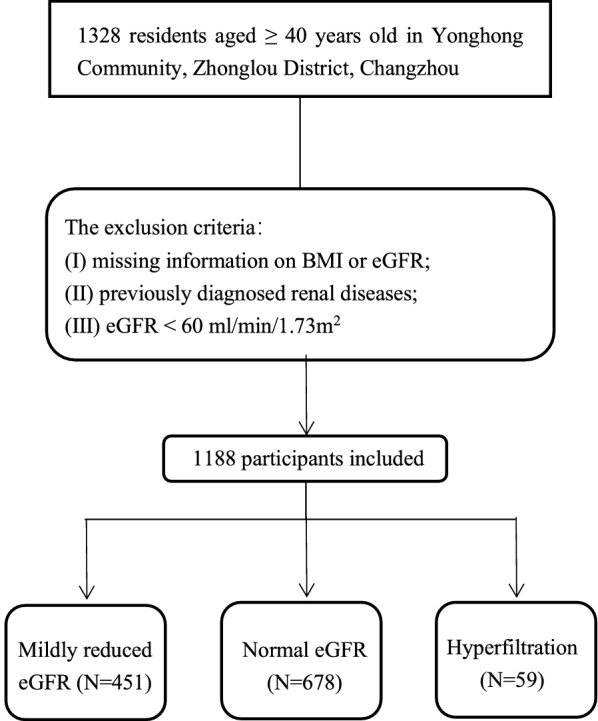
The follow diagram of participants

In the present study, all participants were enrolled from Yonghong Community, Zhonglou District, Changzhou [18, 19]. From December 2016 to December 2017, 1328 residents who had lived in the district for more than 6 months and were 40 years of age or above were recruited. The present analysis excluded participants who had missing body mass index (BMI) or eGFR values, previously diagnosed renal diseases, or an eGFR < 60 ml/min/1.73m^2^. After these exclusions, a total of 1188 participants remained. The flow chart of participants was shown as Fig. 1.

Each participant signed the written informed consent form. The content is that participants provided sociodemographic information and medical history to an interviewer through a standard questionnaire and provided blood samples for biochemical measurements. The study was approved by the Institutional Review Board of the Third Affiliated Hospital of Soochow University.

### Data collection

Trained interviewers obtained information on sociodemographic characteristics, lifestyle factors (including drinking and smoking habits and physical activity) and medical history (including diseases and use of medications) for a detailed series of standardized questions. Current smokers were defined as participants who smoked at least one cigarette per day or seven cigarettes per week, and current drinkers were defined as participants who consumed alcohol at least once per week. Physical activity was categorized as high physical activity or not, according to the International Physical Activity Questionnaire (IPAQ).

Trained staff conducted anthropometric measurements based on standard protocols defined in previous studies [18]. BMI was defined as weight (kg) divided by height squared (m^2^). Blood pressure was measured three times in one-minute intervals, following a five-minute rest (OMRON Model HEM-752 FUZZY, Omron Company, Dalian, China). The average systolic blood pressure (SBP) and average diastolic blood pressure (DBP) were used in the analysis.

After at least a 10-h overnight fast, blood samples were collected for serum lipid profiles, including total cholesterol (TC), triglyceride (TG), high-density lipoprotein cholesterol (HDL-c), low-density lipoprotein cholesterol (LDL-c), liver enzymes (aspartate aminotransferase, AST; alanine aminotransferase, ALT; γ-glutamyl transferase, GGT), serum creatinine, and uric acid (AU-5800 Chemistry System, Beckman, USA). Fasting plasma glucose (FPG) was also measured with an autoanalyzer using the glucose oxidase method (AU-5800 Chemistry System, Beckman, USA). The eGFR values were calculated with the Chronic Kidney Disease Epidemiology Collaboration (CKD-EPI) equations for cystatin C alone: (1) for cystatin C ≤ 0.8 mg/l, eGFR = 133 × (cystatin C/0.8)^−0.449^ × 0.996^age^ × [0.932 if female]; 2) for cystatin C > 0.8 mg/l, eGFR = 133 × (cystatin C/0.8)^−1.328^ × 0.996^age^ × [0.932 if female] [20].

### Definitions

The eGFR was categorized into three groups: mildly reduced eGFR, normal eGFR, and hyperfiltration. A mildly reduced eGFR was defined as eGFR values between 60 and 90 ml/min/1.73m^2^. Hyperfiltration was defined as an eGFR > 95th percentile after adjusting for sex, age, weight and height [21]. Normal eGFR was defined as all eGFR values other than those signaling mildly reduced eGFR and hyperfiltration.

Previous studies have demonstrated that Asian populations have higher percentage of body fat at a given BMI than do white or European populations. Additionally, at the same BMI, Asian populations have higher prevalence of type 2 diabetes and increased cardiovascular risk factors [22]. Thus, we defined obesity as a BMI ≥ 25 kg/m^2^ and non-obesity as a BMI < 25 kg/m^2^ using the World Health Organization Asia Pacific guidelines [23]. We used the Adult Treatment Panel III (ATP III) criteria to define metabolically healthy status as meeting < 2 of the following criteria, excluding waist circumference: (1) elevated SBP (≥ 130 mmHg) and/or DBP (≥ 85 mmHg) or on antihypertensive treatment, (2) high TG (≥ 1.7 mmol/L) or on lipid-lowering medications, (3) high FPG (≥ 5.6 mmol/L) or on medications for diabetes, and 4) low HDL-c (< 1.04 mmol/L in men and < 1.29 mmol/L in women) [16]. The obesity phenotype was defined as: (1) metabolically healthy non-obesity (MHNO): BMI < 25 kg/m^2^ and < 2 metabolic risk factor, (2) MHO: BMI ≥ 25 kg/m^2^ and < 2 metabolic risk factor, (3) metabolically unhealthy nonobesity (MUNO): BMI < 25 kg/m^2^ and ≥ 2 metabolic risk factors, and (4) metabolically unhealthy obesity (MUO): BMI ≥ 25 kg/m^2^ and ≥ 2 metabolic risk factors.

Hyperuricemia was identified in participants in the sex-specific upper quartile of serum uric acid levels (≥ 372 μmol/l for males and ≥ 332 μmol/l for females).

### Statistical methods

Characteristics of the study population were presented according to obesity phenotypes. Means ± standard deviation (SD) and medians (interquartile ranges) were used to describe the normally distributed and skewed continuous variables. Numbers (proportions) were used to present categorical variables. TG, FPG, ALT, AST, and GGT were logarithmically transformed due to their skewed distributions. *P* values for the four obesity phenotypes were calculated using ANOVA for continuous variables and a Chi-squared test for categorical variables. Multiple comparisons among the four groups were made with the Bonferroni test.

We used multivariate-adjusted logistic regression to evaluate the impact of the obesity phenotype on mildly reduced eGFR and hyperfiltration. We established four models as follows: Model 1 was adjusted for age and sex; Model 2 was based on Model 1 and further adjusted for physical activity and current smoking and drinking habits; Model 3 was based on Model 2 and further adjusted for ALT, AST, GGT, TC and LDL-c; and Model 4 was based on Model 3 and further adjusted for uric acid. In addition, we stratified the four obesity phenotypes according to hyperuricemia status (present and not). The Bonferroni method was used to compare the prevalence of mildly reduced eGFR in the hyperuricemia and non-hyperuricemia groups according to obesity phenotype as well as the prevalence of hyperfiltration. With MHNO/non-hyperuricemia as the reference, multivariate logistic regression was used to assess the combined impact of obesity phenotype and uric acid on mildly reduced eGFR.

All statistical analyses were performed with SAS version 9.3 (SAS Institute Inc, Cary, NC). Two-tailed *P* values < 0.05 were considered statistically significant.

## Results

### Characteristics of the study population

Overall, the mean age of total participants was 67.0 ± 8.1 years old, and the proportion of males was 33.6% (n = 399). In all, 35.3% (n = 419) of participants were metabolically healthy and 52.6% (n = 618) were obese. Overall, 152 participants were defined as having MHO, accounting for 12.8% of total participants and 24.6% of obese participants. Table 1 shows the sociodemographic and biochemical characteristics of the study population according to the four obesity phenotypes. Compared to participants in the MHNO group, those with MHO had higher BMI, SBP, and serum uric acid levels; comprised a higher proportion of current drinkers; and had lower levels of HDL-c (all *P* values < 0.05). However, participants in the MHNO and MHO groups had comparable eGFR values. Those who were metabolically unhealthy had higher levels of SBP, TG, and FPG and lower levels of HDL-c, while those with MUNO comprised a lower proportion of current smokers and drinkers than those with MHO (all *P* values < 0.05). Notably, serum uric acid levels were higher in participants with MHO and MUO than in their non-obese counterparts (all *P* values < 0.05). Additionally, characteristics of participants according to renal function were shown in Additional file 1: Table S1.

**Table 1:** Characteristics of study population according to obesity phenotype

Variables	Metabolically healthy		Metabolically unhealthy		*P* value^*^
	Non-obese (MHNO) (n = 267)	Obese (MHO) (n = 152)	Non-obese (MUNO) (n = 303)	Obese (MUO) (n = 466)	
eGFR (ml/min/1.73 m^2^)	96.3 ± 15.0	92.8 ± 15.1	92.7 ± 14.5	91.5 ± 15.1^a^	0.0005
Age (years)	65.8 ± 8.2	67.6 ± 8.4	67.5 ± 8.0	67.1 ± 7.9	0.048
Male, n (%)	85 (31.8)	69 (45.4)	81 (26.7)	164 (35.2)	0.95
BMI (kg/m^2^)	22.2 ± 1.9	27.3 ± 2.1^a^	23.0 ± 1.5^a^^,^^b^	28.1 ± 2.8^a,b,c^	< 0.0001
Lifestyle factors					
Current smokers, n (%)	35 (13.1)	29 (19.1)	28 (9.2)^b^	56 (12.0)	0.24
Current drinkers, n (%)	9 (3.4)	16 (10.5)^a^	8 (2.6)^b^	30 (6.4)	0.40
High physical activity, n (%)	160 (59.9)	90 (59.2)	186 (61.4)	264 (56.7)	0.41
Blood pressure (mmHg)					
SBP	127 ± 14	132 ± 14^a^	136 ± 14^a^	138 ± 13^a,b^	< 0.0001
DBP	81 ± 8	82 ± 9	83 ± 8	85 ± 8^a,b,c^	< 0.0001
Lipid profiles (mmol/L)					
TC	5.26 ± 0.89	5.08 ± 0.96	5.16 ± 1.01	5.04 ± 1.00	0.026
HDL-c	1.57 ± 0.31	1.43 ± 0.25^a^	1.29 ± 0.28^a,b^	1.23 ± 0.24 ^a,b,c^	< 0.0001
LDL-c	2.79 ± 0.68	2.70 ± 0.71	2.74 ± 0.69	2.67 ± 0.66	0.096
TG	1.15 (0.89–1.45)	1.34 (1.05–1.51)	1.86 (1.41–2.43)^a,b^	1.98 (1.52–2.61) ^a,b^	< 0.0001
Liver enzymes (U/L)					
AST	22.0 (19.0–26.0)	22.0 (20.0–27.0)	22.0 (19.0–27.0)	24.0 (19.0–29.0)	0.012
ALT	16.0 (13.0–22.0)	18.0 (14.0–23.0)	18.0 (14.0–24.0)	22.0 (16.0–31.0)^a,c^	< 0.0001
GGT	17.0 (13.0–25.0)	19.0 (15.0–28.0)	18.0 (15.0–29.0)	24.5 (18.0–33.0)^a^	0.004
FPG (mmol/L)	4.87 (4.49–5.44)	5.16 (4.63–5.60)	6.13 (4.99–7.95) ^a,b^	6.37 (5.22–7.38)^a,b^	< 0.0001
Serum uric acid (μmol/L)	278 ± 65	308 ± 79^a^	292 ± 64	321 ± 71^a,c^	< 0.0001

Data were presented as means ± SD for median (interquartile ranges) for continuous variables, and numbers (proportions) for categorical variables

*eGFR* estimated glomerular filtration rate; *BMI* body mass index; *SBP* systolic blood pressure; *DBP* diastolic blood pressure; *TC* total cholesterol; *HDL-c* high-density lipoprotein cholesterol; *LDL-c* low-density lipoprotein cholesterol; *TG* triglyceride; *AST* aspartate aminotransferase; *ALT* alanine aminotransferase; *GGT* γ-glutamyltransferase; *FPG* fasting plasma glucose

^*^ANOVA for continuous variables and Chi-square test for categorical variables were used to assess the differences among the four groups

^a^Compared with MHNO, *P* < 0.05 calculated by Bonferroni test

^b^Compared with MHO, *P* < 0.05 calculated by Bonferroni test

^c^Compared with MUNO, *P* < 0.05 calculated by Bonferroni test

### Obesity phenotype and the risk of mildly reduced eGFR and hyperfiltration

**Fig. 2:**
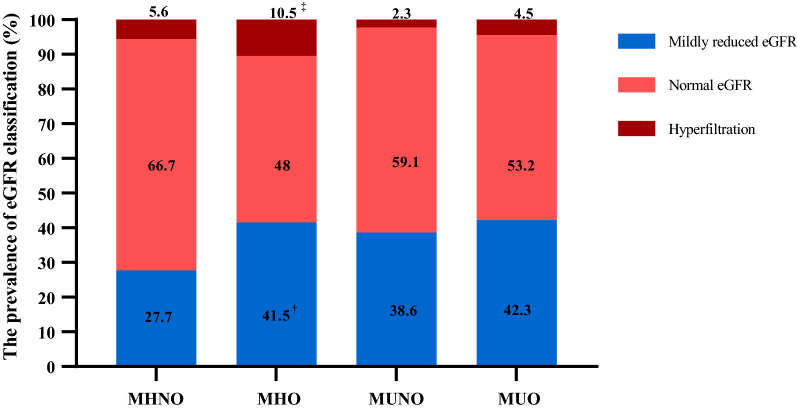
The prevalence of mildly reduced eGFR, normal eGFR and hyperfiltration according to obesity phenotype. The Bonferroni test was used for multiple comparisons among the four phenotypes, ^†^*P* < 0.05, the prevalence of mildly reduced eGFR in MHO compared to MHNO; ^‡^*P* < 0.05, the prevalence of hyperfiltration in MHO compared to MUNO; *eGFR* estimated glomerular filtration rate; *MHNO* Metabolically healthy non-obesity; *MHO* Metabolically healthy obesity; *MUNO* Metabolically unhealthy non-obesity; *MUO* Metabolically unhealthy obesity

Figure 2 shows the prevalence of mildly reduced eGFR, normal eGFR, and hyperfiltration according to four obesity phenotypes. The crude prevalences of mildly reduced eGFR and hyperfiltration were 27.7% and 5.6% for the MHNO phenotype, 41.5% and 10.5% for the MHO phenotype, 38.6% and 2.3% for the MUNO phenotype, and 42.3% and 4.5% for the MUO phenotype. The Bonferroni test for multiple comparisons among the four phenotypes showed that the prevalence of mildly reduced eGFR in individuals with MHO was higher than individuals with MHNO (*P* = 0.0026, 41.5% *vs.* 27.7%), but not statistically different from the prevalence in metabolically unhealthy individuals (*P* > 0.05). Conversely, the Bonferroni test found that the crude prevalence of hyperfiltration in individuals with MHO was higher than in individuals in the MUNO group (*P* = 0.002, 10.5% *vs.* 2.3%), but found no statistical difference between the MHO and MHNO phenotypes (*P* > 0.05).

Multinomial logistic regression revealed an association between the MHO phenotype and prevalent mildly reduced eGFR (Table 2). The multi-adjusted odds ratio (OR) for mildly reduced eGFR associated with MHO was 1.85 (95% CI 1.13–3.01), referenced to MHNO (Model 3 in Table 2). However, adjusting for uric acid reduced the association between the MHO phenotype and prevalent mildly reduced eGFR to null (OR = 1.49, 95% CI 0.90–2.48) (Model 4 in Table 2). Analogously, the MUNO and MUO phenotypes were significantly associated with a higher risk of mildly reduced eGFR after adjusting for confounding factors (Model 3 in Table 2). Adjusting for uric acid resulted in the disappearance of the associations (Model 4 in Table 2). In addition, the MHO phenotype had a 1.28-fold higher risk of hyperfiltration compared to the nonobese counterparts (OR = 2.28, 95% CI 1.03–5.09) with multiple adjustments. After further adjusting for uric acid, the risk of hyperfiltration still remained significant (OR = 2.57, 95% CI 1.14–5.77). In contrast, there were no significant associations found between hyperfiltration and metabolically unhealthy individuals.

**Table 2:** Odds ratios of mildly reduced eGFR and hyperfiltration according to obesity phenotype

	Metabolically healthy		Metabolically unhealthy	
	Non-obese (MHNO)	Obese (MHO)	Non-obese (MUNO)	Obese (MUO)
For mildly reduced eGFR				
Case/number (%)	74/267 (27.7)	63/152 (41.5)	117/303 (38.6)	197/466 (42.3)
Model 1 (adjusted for age and sex)	1.00 (Ref.)	1.77 (1.10–2.86)	1.42 (0.95–2.11)	1.80 (1.25–2.59)
Model 2 (further adjusted for physical activity, current smoking and drinking habits)	1.00 (Ref.)	1.85 (1.14–3.00)	1.42 (0.95–2.12)	1.86 (1.29–2.68)
Model 3 (further adjusted for ALT, AST, GGT, TC and LDL-c)	1.00 (Ref.)	1.85 (1.13–3.01)	1.51 (1.01–2.27)	2.00 (1.35–2.96)
Model 4 (further adjusted for uric acid)	1.00 (Ref.)	1.49 (0.90–2.48)	1.36 (0.89–2.06)	1.46 (0.97–2.19)
For hyperfiltration				
Case/number (%)	15/267 (5.6)	16/152 (10.5)	7/303 (2.3)	21/466 (4.5)
Model 1 (adjusted for age and sex)	1.00 (Ref.)	2.11 (0.97–4.61)	0.45 (0.18–1.16)	0.95 (0.47–1.92)
Model 2 (further adjusted for physical activity, current smoking and drinking habits)	1.00 (Ref.)	2.33 (1.06–5.13)	0.45 (0.17–1.14)	0.99 (0.49–2.01)
Model 3 (further adjusted for ALT, AST, GGT, TC and LDL-c)	1.00 (Ref.)	2.28 (1.03–5.09)	0.42 (0.16–1.10)	0.93 (0.44–1.97)
Model 4 (further adjusted for uric acid)	1.00 (Ref.)	2.57 (1.14–5.77)	0.40 (0.15–1.06)	1.11 (0.51–2.40)

*eGFR* estimated glomerular filtration rate; *TC* total cholesterol; *LDL-c* low-density lipoprotein cholesterol; *AST* aspartate aminotransferase; *ALT* alanine aminotransferase; *GGT* γ-glutamyltransferase; *MHNO* Metabolically healthy non-obesity; *MHO* Metabolically healthy obesity; *MUNO* Metabolically unhealthy non-obesity; *MUO* Metabolically unhealthy obesity

Model 1: adjusted for age and sex;

Model 2: further adjusted for physical activity, current smokers (yes/no), current drinking (yes/no) on basis of model 1;

Model 3: further adjusted for ALT, AST, GGT, TC and LDL-c on basis of model 2;

Model 4: further adjusted for the level of uric acid on basis of model 3

### Combined effects of obesity phenotype and uric acid on the risk of mildly reduced eGFR and hyperfiltration

**Fig. 3:**
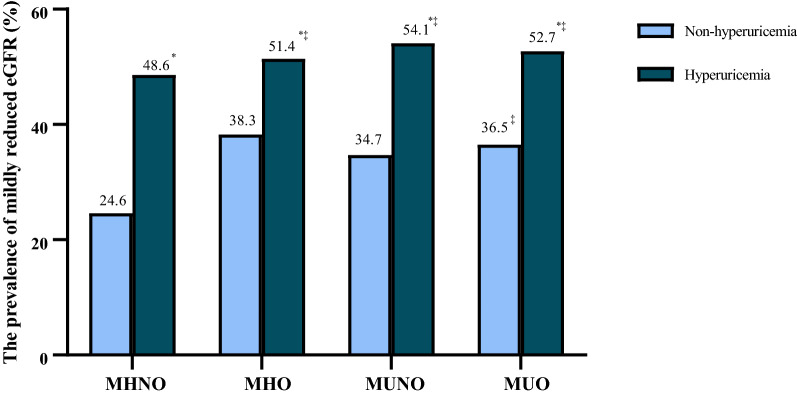
The prevalence of mildly reduced eGFR according to obesity phenotype and serum uric acid level. *P* values were calculated by Bonferroni method, ^*^P < 0.05, each obesity phenotype with hyperuricemia *vs.* non-hyperuricemia counterparts; ^‡^ *P* < 0.05 the other groups vs. MHNO/non-hyperuricemia group. *eGFR* estimated glomerular filtration rate; *MHNO* Metabolically healthy non-obesity; *MHO* Metabolically healthy obesity; *MUNO* Metabolically unhealthy non-obesity; *MUO* Metabolically unhealthy obesity

**Fig. 4:**
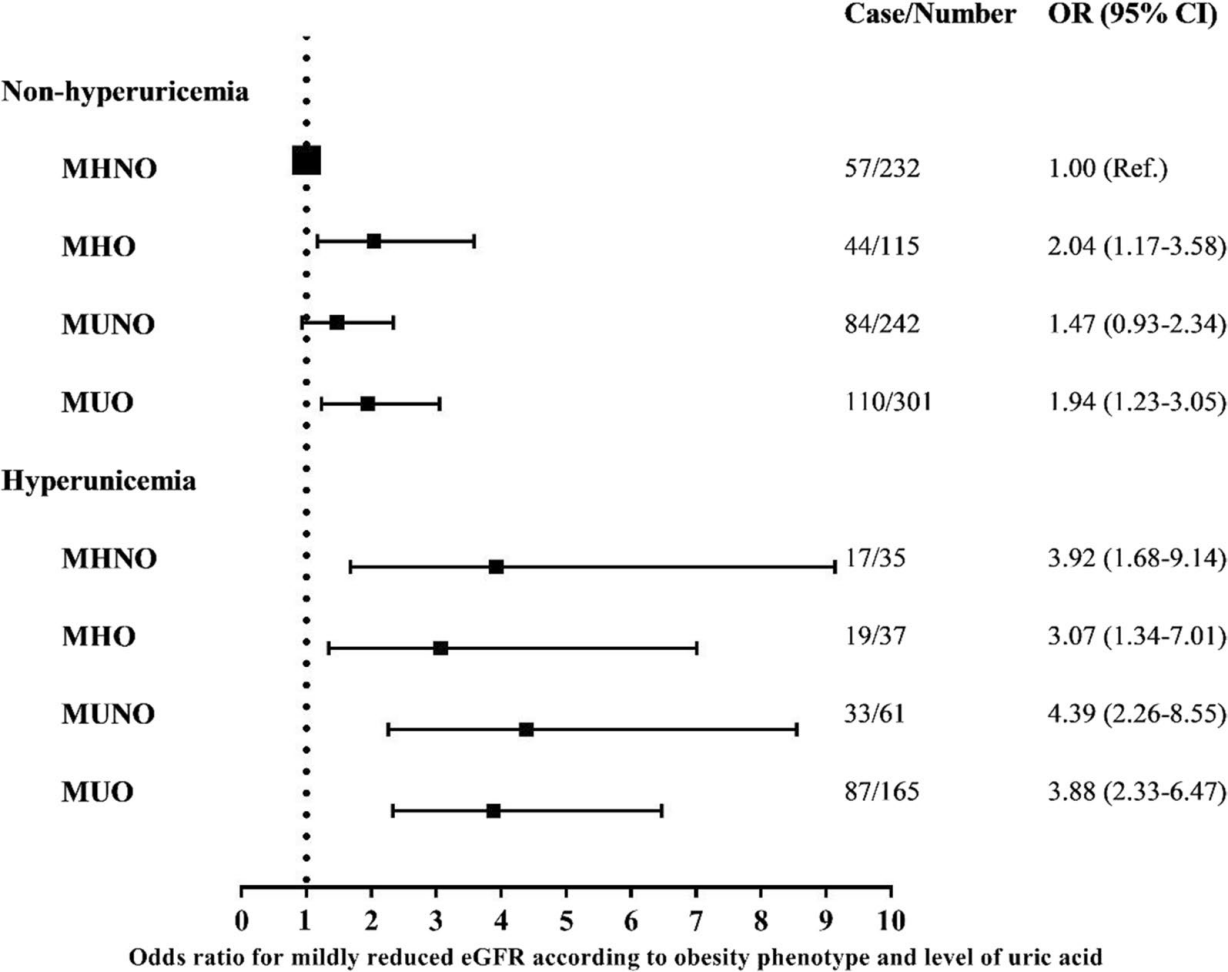
Odds ratio of mildly reduced eGFR according to obesity and the level of serum uric acid. Odds ratios were calculated by multivariable logistic regression adjusting for age, sex, physical activity, current smokers (yes/no), current drinking (yes/no), ALT, AST, GGT, TC and LDL-c. *eGFR* estimated glomerular filtration rate; *MHNO* Metabolically healthy non-obesity; *MHO* Metabolically healthy obesity; *MUNO* Metabolically unhealthy non-obesity; *MUO* Metabolically unhealthy obesity. *OR* Odds ratio

Figure 3 shows that the MHNO/non-hyperuricemia group had the lowest prevalence (24.6%), and the MUO/hyperuricemia group had the highest prevalence (52.7%), of mildly reduced eGFR. The Bonferroni test further found that the four obesity phenotypes with hyperuricemia had markedly higher prevalences of mildly reduced eGFR than the four corresponding non-hyperuricemia groups (All *P* values < 0.05). Figure 4 displays the multivariate-adjusted ORs of mildly reduced eGFR according to obesity phenotype and serum uric acid level. Compared to the MHNO group with non-hyperuricemia, the MHO group with non-hyperuricemia had a 1.04-fold higher risk of mildly reduced eGFR (OR = 2.04, 95% CI 1.17–3.58). In addition, the MHO group with hyperuricemia had an observably increased risk (OR = 3.07, 95% CI 1.34–7.01). The two metabolically unhealthy phenotypes were associated with an increased risk of mildly reduced eGFR, irrespective of the level of uric acid, except for the MUNO group with non-hyperuricemia. Moreover, we performed stratified analyses using MHNO in each uric acid group as the reference and found that the MHO phenotype and the metabolically unhealthy phenotypes with hyperuricemia no longer had a higher risk of mildly reduced eGFR, while the MHO and MUO phenotypes with non-hyperuricemia remained the significantly higher risk of mildly reduced eGFR (data shown in Additional file 2: Table S2).

We also assessed the combined effect of obesity phenotype and uric acid on hyperfiltration (Additional file 4: Fig. S1). Non-obese individuals were non-hyperuricemic, irrespective of metabolic status. The prevalence of hyperfiltration in the MHNO/non-hyperuricemia and MUNO/non-hyperuricemia groups were 6.5% and 2.9%, respectively. The MHO/non-hyperuricemia group did not have a statistically higher prevalence of hyperfiltration than its hyperuricemia counterpart (12.2% *vs.* 5.4%, *P* > 0.05), nor did it have a higher prevalence than the MUO/non-hyperuricemia group (5.7% *vs.* 2.4%, *P* > 0.05). Moreover, the MHNO/non-hyperuricemia group’s prevalence of hyperfiltration was not markedly different from that of any other group across the obesity phenotypes and uric acid levels (all *P* values > 0.05). However, multivariate logistic regression revealed that when referenced to the MHNO/non-hyperuricemia phenotype, a 139% increased risk of hyperfiltration was found in the MHO/non-hyperuricemia phenotype (OR = 2.39, 95% CI = 1.04–5.52), whereas the increased risk was not found in MHO/Hyperuricemia phenotype. The MUNO and MUO phenotypes did not have a significant association with prevalent hyperfiltration, with or without hyperuricemia present (Additional file 3: Table S3).

## Discussion

In this study, we found that obesity without metabolic abnormalities was adversely associated with early kidney dysfunction among middle-aged and elderly Chinese adults. Participants with MHO had a significantly higher risk of hyperfiltration and mildly reduced eGFR compared to the MHNO phenotype. Furthermore, we found that the significant association between MHO and mildly reduced eGFR attenuated to null after adjusting for serum uric acid level. Additionally, we found that different levels of serum uric acid affect the association between MHO and mildly reduced eGFR. These findings clarified that obesity per se increases the risk of early kidney dysfunction and metabolically healthy status cannot prevent obese patients from early kidney dysfunction. Interestingly, hyperuricemia might partly explain the association of MHO with mildly reduced eGFR.

Obesity has been considered a critical risk factor for chronic diseases, such as cardiovascular disease and mortality. Recently, an obesity paradox phenomenon has been reported among CKD patients [24], in which higher BMI levels are paradoxically associated with greater survival and lower BMI levels are associated with higher mortality, especially in ESRD patients [25, 26]. The potential mechanisms of the obesity paradox are indeterminate and may be partly attributed to discrepancies in metabolic status among obese patients. Better metabolic reserves may prevent obese patients from unfavorable metabolic dysregulation as compared to their non-obese counterparts [27]. Thus, we took metabolically healthy status into account in our obesity phenotypes. MHO is characterized by obesity without metabolic abnormalities and is considered to protect obese patients from the metabolic complications of obesity; furthermore, the risks of chronic diseases appear to be lower than that of the given BMI [28].

However, could the MHO phenotype actually indicate a favorable prognosis? In terms of type 2 diabetes [29], subclinical cardiovascular disease (CVD) [30], and long-term CVD risk [31, 32], the answer to this question has been widely debated in the literature. Meanwhile, individuals with MHO may be heterogeneous with respect to CKD. Recently, a retrospective cohort study based on a British primary care population declared that metabolically healthy overweight and MHO individuals had 1.30-fold and 1.66-fold risks of incident CKD compared to those with MHNO, respectively [15]. Another prospective study conducted in middle-aged and elderly Chinese adults found that those with MHO had a 65% increased risk for incident CKD than those with MHNO [16]. Our results, which focused on MHO and early kidney dysfunction, discovered the same trends as these studies. Previous studies and our study suggest that individuals with MHO, regardless of ethnicity, have an increased risk of early and advanced kidney dysfunction. However, several studies have refuted these conclusions. The Reasons for Geographic and Racial Differences in Stroke (REGARDS) study enrolled 30,239 Black and white adults and followed up for 6.3 years, reported that a higher BMI was associated with a lower risk of ESRD in those without metabolic syndrome (hazard ratio was 0.70 (0.52–0.95) per 5 kg/m^2^ increase in BMI) but not in those with metabolic syndrome [33]. The different results from our study could be attributed to the study population and the definition of metabolic health. Most participants in the REGARDS study were African Americans aged ≥ 45 years, and the study included waist circumference in its definition of metabolically healthy status, while our study was conducted in a Chinese population and excluded waist circumference in the definition of metabolically healthy status. Alternatively, two prospective studies conducted in Asia found that metabolic health mediated the association between obesity and CKD and that the MHO phenotype was not associated with a higher risk of incident CKD [17, 34]. Younger participants, advanced kidney dysfunction and dissimilar definitions of eGFR may have contributed to the discrepancies with our study. In these two studies, the primary outcome was incident CKD defined as serum-creatinine-based eGFR < 60 ml/min/1.73m^2^. In contrast, the main outcome in our study was early kidney dysfunction-hyperfiltration and mildly reduced eGFR calculated with serum cystatin C, which is an earlier biomarker of kidney dysfunction.

The current study is the first to investigate the associations of MHO with both hyperfiltration and mildly reduced eGFR. We found that individuals with MHO had a 2.28-fold increase in risk of hyperfiltration and an 85% increased risk of mildly reduced eGFR relative to MHNO individuals. Our results suggest that obesity is not harmless in terms of kidney dysfunction, even at an early stage. An analogous conclusion was also drawn by the Korean National Health and Nutrition Examination Survey, in which MHO indicated a 49% increased risk of microalbuminuria compared to MHNO [35].

The association between obesity phenotype and early kidney dysfunction might be mediated by multiple mechanisms. Insulin resistance [36], chronic inflammation [16], and visceral adiposity [37] have been implicated in previous studies. In addition, uric acid might play a crucial role on kidney injury in obese patients. Prospective studies found that elevated baseline uric acid levels were associated with an increased risk of CKD and that changes in uric acid levels were inversely associated with renal function decline [38, 39]. Additionally, in individuals without metabolic abnormalities, increased uric acid levels have been strongly associated with albuminuria [40]. In accordance with these findings, we observed that the association of MHO with mildly reduced eGFR was statistically attenuated through adjustment for the uric acid level. Referenced to MHNO with non-hyperuricemia, the OR of mildly reduced eGFR was 2.04 (95% CI = 1.17–3.58) for MHO with non-hyperuricemia and 3.07 (95% CI = 1.34–7.01) for MHO with hyperuricemia. Furthermore, we did not find an increased risk of mildly reduced eGFR in MHO with hyperuricemia when referenced to MHNO with hyperuricemia, whereas MHO with non-hyperuricemia still had a 1.13-fold higher risk of mildly reduced eGFR when referenced to MHNO with non-hyperuricemia. The results imply that uric acid levels partly mediate the association between MHO and early kidney dysfunction, especially in individuals with hyperuricemia. Additional longitudinal studies are needed to elucidate the conclusive association between MHO and early kidney dysfunction and to determine the modifying effects of uric acid on this association.

As a cross-sectional study, this study had unavoidable limitations of note. First, the causal relationship between MHO and early kidney dysfunction could not be inferred. Hence, further prospective studies are warranted to clarify the role of MHO on the progression of kidney dysfunction. Second, early kidney dysfunction was defined based on eGFR ( via the CKD-EPI equation) rather than directly measured. However, in a large epidemiological study, the CKD-EPI equation was used as a standard method of estimating GFR and were demonstrated to be better than the most frequently used Modification of Diet in Renal Disease (MDRD) equation in the estimation of GFR for both CKD and healthy individuals [41]. We would further measured the proteinuria and albuminuria levels to evaluate renal function of participants in longitudinal study. Third, our study’s focus on middle-aged and elderly Chinese adults limited the generalizability of the results to other ethnic groups. Fourth, we didn’t measure insulin levels and inflammation-related markers of participants in present study. We could not directly evaluate the influence of insulin resistance and inflammation on the association. However, we instead used the triglyceride-glucose (TyG) index to evaluate insulin resistance[42]. We further adjusted TyG index based on Model 3 in Table 2. Further adjusting TyG index caused little changes and the significant relationship still remained. Hence, we speculated that the insulin resistance of participants has no significant influence on the association of obesity phenotype with renal function. Finally, information on diet and urate-lowering therapy was not collected in the present study. A follow-up study would need to be designed to acquire this information.

## Conclusions

In conclusion, among the middle-aged and elderly Chinese population, we found that MHO was associated with an increased risk of early kidney dysfunction, including hyperfiltration and mildly reduced eGFR. Our results suggest that MHO is not a harmless condition and that a metabolically healthy status cannot protect obese patients from early kidney dysfunction. Additionally, we found that the significant association between MHO and mildly reduced eGFR attenuated to null after adjusting for serum uric acid level and that the association between MHO and mildly reduced eGFR was not significant among those with hyperuricemia. These results suggest that uric acid partially explains the association of MHO with mildly reduced eGFR, especially in individuals with hyperuricemia. Hence, uric acid should be taken into account when estimating the risk of early kidney dysfunction in individuals with MHO. Prospective studies are warranted to clarify the causality in the future.

## Supplementary Information

**Additional file 1: Table S1.** Characteristics of study population according to renal function.

**Additional file 2: Table S2.** Odds ratios for mildly reduced eGFR according to obesity phenotype and serum uric acid level (MHNO as reference in each uric acid level).

**Additional file 3: Table S3.** Odds ratio (95% CI) for hyperfiltration according to obesity phenotype and serum uric acid level.

**Additional file 4:** **Figure S1.** The prevalence of hyperfiltration according to obesity phenotype and the serum uric acid level.

## Data Availability

The data in the current study arisen from a dataset of Department of Endocrine and Metabolic Diseases, the First People’s Hospital of Changzhou, Third Affiliated Hospital of Soochow University, are not publicly available due to security consideration, but are available from the corresponding author on reasonable request.

## Abbreviations

AST: Aspartate aminotransferase
ALT: Alanine aminotransferase
ATP III: Adult Treatment Panel III
BMI: Body mass index
CKD: Chronic kidney disease
CVD: Cardiovascular disease
CKD-EPI: Chronic Kidney Disease Epidemiology Collaboration
CI: Confidence interval
DBP: Diastolic blood pressure
ESRD: End-stage renal disease
FPG: Fasting plasma glucose
eGFR: Estimated glomerular filtration rate
GH: Glomerular hyperfiltration
GFR: Glomerular filtration rate
GGT: γ-Glutamyl transferase
HDL-c: High-density lipoprotein cholesterol
IPAQ: International Physical Activity Questionnaire
LDL-c: Low-density lipoprotein cholesterol
MHO: Metabolically healthy obesity
MHNO: Metabolically healthy non-obesity
MUNO: Metabolically unhealthy non-obesity
MUO: Metabolically unhealthy obesity
OR: Odds ratio
REGARDS: The Reasons for Geographic and Racial Differences in Stroke
SBP: Systolic blood pressure
SD: Standard deviation
TC: Total cholesterol
TG: Triglyceride
