# Supplementary material for: Metabolically healthy obesity is associated with higher risk of both hyperfiltration and mildly reduced estimated glomerular filtration rate: the role of serum uric acid in a cross-sectional study
Source: J Transl Med. 2023 Mar 23;21:216. doi: 10.1186/s12967-023-04003-y (PMC10035285; doi:10.1186/s12967-023-04003-y)
Supplement: Supplementary file 1 — Additional file 1: Table S1. Characteristics of study population according to renal function. [file 12967_2023_4003_MOESM1_ESM.docx]

| **Table S1. Characteristics of study population according to renal function** | | | | |
| --- | --- | --- | --- | --- |
| **Variables** | **Mildly reduced eGFR** | **Normal eGFR** | **Hyperfiltration** | ***P* values for trend^*^** |
| eGFR (ml/min/1.73 m^2^) | 77.0 ± 8.2 | 101.9 ± 7.4 | 114.7 ± 7.7 | <0.0001 |
| Serum creatinine (μmol/L) | 79.9 ± 13.0 | 69.4 ± 10.8 | 67.1 ± 12.6 | <0.0001 |
| Age (years) | 71.1 ± 6.4 | 64.0 ± 8.0 | 69.7 ± 5.1 | <0.0001 |
| Male, n (%) | 179 (39.7) | 186 (27.4) | 34 (57.6) | 0.14 |
| BMI (kg/m^2^) | 25.8 ± 3.6 | 25.1 ± 3.3 | 25.8 ± 3.8 | 0.0076 |
| **Lifestyle factors** | | | | |
| Current smokers, n (%) | 57 (12.6) | 83 (12.2) | 8 (13.6) | 0.97 |
| Current drinkers, n (%) | 23 (5.1) | 37 (5.5) | 3 (5.1) | 0.86 |
| High physical activity, n (%) | 268 (59.4) | 391 (57.7) | 41 (69.5) | 0.67 |
| **Blood pressure (mmHg)** | | | | |
| SBP | 136 ± 14 | 133 ± 14 | 131 ± 13 | <0.0001 |
| DBP | 82 ± 9 | 83 ± 8 | 83 ± 9 | 0.827 |
| **Lipid profiles (mmol/L)** | | | | |
| TC | 5.03 ± 1.02 | 5.19 ± 0.94 | 5.09 ± 0.97 | 0.0313 |
| HDL-C | 1.30 ± 0.29 | 1.37 ± 0.30 | 1.43 ± 0.28 | <0.0001 |
| LDL-C | 2.64 ± 0.68 | 2.76 ± 0.67 | 2.77 ± 0.73 | 0.0061 |
| TG | 1.60 (1.18-2.20) | 1.62 (1.18-2.25) | 1.45 (1.15-1.73) | 0.3455 |
| **Liver enzymes (U/L)** | | | | |
| AST | 24.0 (20.0-28.0) | 22.00 (19.0-27.0) | 23.00 (18.0-27.0) | 0.0002 |
| ALT | 19.0 (14.7-26.0) | 19.00 (14.0-26.0) | 18.00 (15.0-26.0) | 0.8609 |
| GGT | 21.0 (16.0-30.0) | 20.00 (15.0-29.0) | 20.00 (15.0-28.0) | 0.2929 |
| FPG (mmol/L) | 5.56 (4.81-6.81) | 5.53 (4.79-6.85) | 5.99 (5.01-7.12) | 0.5210 |
| Serum uric acid (μmol/L) | 326 ± 74 | 290 ± 65 | 268 ± 66 | <0.0001 |
| Data were presented as means ± SD for median (interquartile ranges) for continuous variables, and numbers (proportions) for categorical variables.  ^*^ *P* values for trend across the three groups were calculated using linear regression for continuous variables and Chi-square test for categorical variables.  Abbreviations: eGFR, estimated glomerular filtration rate; BMI, body mass index; SBP, systolic blood pressure; DBP, diastolic blood pressure; TC, total cholesterol; HDL-c, high-density lipoprotein cholesterol; LDL-c, low-density lipoprotein cholesterol; TG, triglyceride; AST, aspartate aminotransferase; ALT, alanine aminotransferase; GGT, γ-glutamyltransferase; FPG, fasting plasma glucose. | | | | |
