# Supplementary material for: Metabolically healthy obesity is associated with higher risk of both hyperfiltration and mildly reduced estimated glomerular filtration rate: the role of serum uric acid in a cross-sectional study
Source: J Transl Med. 2023 Mar 23;21:216. doi: 10.1186/s12967-023-04003-y (PMC10035285; doi:10.1186/s12967-023-04003-y)
Supplement: Supplementary file 2 — Additional file 2: Table S2. Odds ratios for mildly reduced eGFR according to obesity phenotype and serum uric acid level (MHNO as reference in each uric acid level). [file 12967_2023_4003_MOESM2_ESM.docx]

| **Table S2. Odds ratios for mildly reduced eGFR according to obesity phenotype and serum uric acid level (MHNO as reference in each uric acid level)** | | | | |
| --- | --- | --- | --- | --- |
|  | **Metabolically healthy** | | **Metabolically unhealthy** | |
|  | **Non-obese**  **(MHNO)** | **Obese**  **(MHO)** | **Non-obese**  **(MUNO)** | **Obese**  **(MUO)** |
| **Model 1** | | | | |
| Non-hyperuricemia | 1.00 (Ref.) | 1.95 (1.12-3.39) | 1.35 (0.86-2.14) | 1.73 (1.12-2.66) |
| Hyperuricemia | 1.00 (Ref.) | 0.85 (0.30-2.38) | 1.11 (0.45-2.75) | 0.98 (0.44-2.19) |
| **Model 2** | | | | |
| Non-hyperuricemia | 1.00 (Ref.) | 2.07 (1.18-3.63) | 1.36 (0.86-2.16) | 1.76 (1.14-2.71) |
| Hyperuricemia | 1.00 (Ref.) | 0.77 (0.27-2.23) | 1.04 (0.42-2.58) | 0.94 (0.41-2.14) |
| **Model 3** | | | | |
| Non-hyperuricemia | 1.00 (Ref.) | 2.13 (1.20-3.78) | 1.51 (0.94-2.43) | 2.04 (1.28-3.27) |
| Hyperuricemia | 1.00 (Ref.) | 0.73 (0.25-2.16) | 1.06 (0.41-2.75) | 0.96 (0.40-2.30) |

Model 1: adjusted for age and sex;

Model 2: further adjusted for physical activity, current smokers (yes/no), current drinking (yes/no) on basis of model 1;

Model 3: further adjusted for ALT, AST, GGT, TC and LDL-c on basis of model 2;

Abbreviations: eGFR, estimated glomerular filtration rate; TC, total cholesterol; LDL-c, low-density lipoprotein cholesterol; AST, aspartate aminotransferase; ALT, alanine aminotransferase; GGT, γ-glutamyltransferase.
