# Supplementary material for: Metabolically healthy obesity is associated with higher risk of both hyperfiltration and mildly reduced estimated glomerular filtration rate: the role of serum uric acid in a cross-sectional study
Source: J Transl Med. 2023 Mar 23;21:216. doi: 10.1186/s12967-023-04003-y (PMC10035285; doi:10.1186/s12967-023-04003-y)
Supplement: Supplementary file 3 — Additional file 3: Table S3. Odds ratio (95% CI) for hyperfiltration according to obesity phenotype and serum uric acid level. [file 12967_2023_4003_MOESM3_ESM.docx]

| Table S3. Odds ratio (95% CI) for hyperfiltration according to obesity phenotype and serum uric acid level. | | |
| --- | --- | --- |
|  | Non-hyperuricemia | Hyperuricemia |
| MHNO | 1.00 (Ref.) | - |
| MHO | 2.39 (1.04-5.52) | 1.23 (0.24-6.16) |
| MUNO | 0.44 (0.17-1.14) | - |
| MUO | 0.98 (0.44-2.15) | 0.58 (0.18-1.91) |

Odds ratios were calculated by multivariable logistic regression adjusting for age, sex, physical activity, current smokers (yes/no), current drinking (yes/no), ALT, AST, GGT, TC and LDL-c.

Abbreviations: TC, total cholesterol; LDL-c, low-density lipoprotein cholesterol; AST, aspartate aminotransferase; ALT, alanine aminotransferase; GGT, γ-glutamyltransferase.
