# Supplementary material for: Metabolically healthy obesity is associated with higher risk of both hyperfiltration and mildly reduced estimated glomerular filtration rate: the role of serum uric acid in a cross-sectional study
Source: J Transl Med. 2023 Mar 23;21:216. doi: 10.1186/s12967-023-04003-y (PMC10035285; doi:10.1186/s12967-023-04003-y)
Supplement: Supplementary file 4 — Additional file 4: Figure S1. The prevalence of hyperfiltration according to obesity phenotype and the serum uric acid level. [file 12967_2023_4003_MOESM4_ESM.pdf]

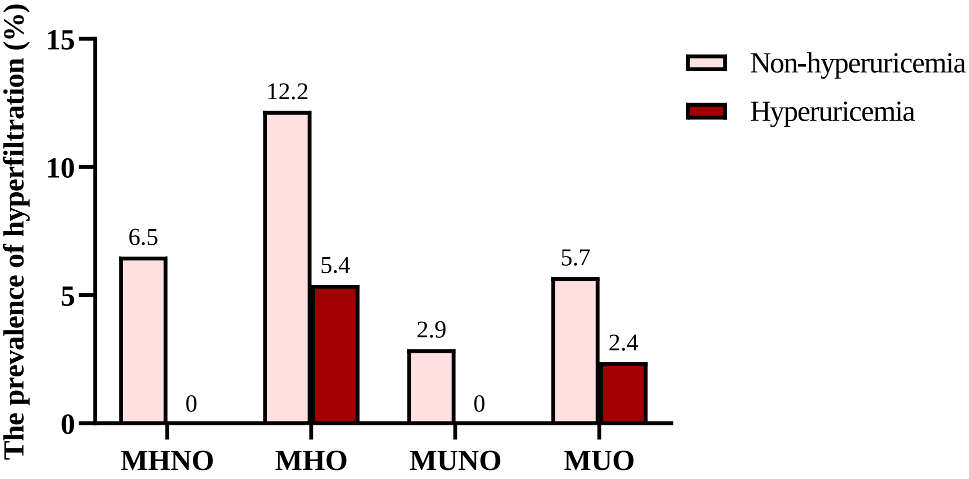

Supplementary Figure 1. The prevalence of hyperfiltration according to obesity phenotype and the serum uric acid level.

All *P* values of multiple comparisons among groups according to obesity phenotypes and serum uric acid were >0.05, using Bonferroni test.

Abbreviations: MHNO, Metabolically healthy non-obesity; MHO, Metabolically healthy obesity; MUNO, Metabolically unhealthy non-obesity; MUO, Metabolically unhealthy obesity.
